# Tumor Growth Rate Decline despite Progressive Disease May Predict Improved Nivolumab Treatment Outcome in mRCC: When RECIST Is Not Enough

**DOI:** 10.3390/cancers13143492

**Published:** 2021-07-12

**Authors:** Veronica Mollica, Stefano Brocchi, Filippo Gustavo Dall’Olio, Laura Marcolin, Alexandro Paccapelo, Matteo Santoni, Alessandro Rizzo, Rodolfo Montironi, Rita Golfieri, Francesco Massari, Andrea Ardizzoni

**Affiliations:** 1 Medical Oncology, IRCCS Azienda Ospedaliero-Universitaria di Bologna, Via Albertoni n. 15, 40138 Bologna, Italy; veronica.mollica2@unibo.it (V.M.); filippo.dallolio4@unibo.it (F.G.D.); alessandro.rizzo11@studio.unibo.it (A.R.); andrea.ardizzoni@aosp.bo.it (A.A.); 2 Department of Radiology, IRCCS Azienda Ospedaliero-Universitaria di Bologna, Via Albertoni 15, 40138 Bologna, Italy; stefano.brocchi@aosp.bo.it (S.B.); laura.marcolin@studio.unibo.it (L.M.); alexandro.paccapelo@aosp.bo.it (A.P.); rita.golfieri@aosp.bo.it (R.G.); 3 Oncology Unit, Macerata Hospital, 62100 Macerata, Italy; mattymo@alice.it; 4 Section of Pathological Anatomy, Polytechnic University of the Marche Region, School of Medicine, United Hospitals, 60126 Ancona, Italy; r.montironi@staff.univpm.it

**Keywords:** renal cell carcinoma, RCC, nivolumab, immunotherapy, tumor growth rate, TGR, RECIST

## Abstract

**Simple Summary:**

The treatment scenario of metastatic renal cell carcinoma has drastically changed in recent years, with the advent of immunotherapy. Since 2015 immune-checkpoint inhibitors, either alone or in combination with other compounds, are constantly enriching the treatment scenario, with a drastic change of patients’ outcomes. The benefit from immunotherapy is difficult to capture with the currently available assessment radiological criteria. Often, with rhe use of immunotherapy, we can observe atypical patterns of response, such as hyperprogression or pseudoprogression. Pseudoprogression consists of an initial increase in tumor burden followed by a response to therapy, while hyperprogression is defined as a tumor growth rate that was at least 2-fold greater in patients with disease progression during immunotherapy. We performed a retrospective monocentric study to explore the impact of tumor growth rate change after immunotherapy administration as second or later line of treatment in patients with metastatic renal cell carcinoma.

**Abstract:**

Treatment response is usually assessed by the response evaluation criteria in solid tumors (RECIST). These criteria may not be adequate to evaluate the response to immunotherapy, considering the peculiar patterns of response reported with this therapy. With the advent of immunotherapy these criteria have been modified to include the evaluation of the peculiar responses seen with this type of therapy (iRECIST criteria), including pseudoprogressions and hyperprogressions. Tumor growth rate (TGR) is a dynamic evaluation that takes into account the kinetics of response to treatment and may help catch the real efficacy of an immunotherapy approach. We performed a retrospective monocentric study to explore the impact of TGR change after nivolumab administration as the second or later line of treatment in patients with metastatic renal cell carcinoma (RCC). We evaluated 27 patients, divided into three categories: Disease control (DC) if there was no PD; lower velocity PD (LvPD) if disease progressed but the TGR at second assessment (TGR2) was lower than the TGR at first assessment (TGR1); higher velocity PD (HvPD) if TGR2 was higher than TGR1. The median OS for the DC group was 11.0 months (95% CI 5.0–17.0) (reference) vs. (not reached) NR (95% CI NR-NR) for LvPD (HR 0.27; 95% CI 0.06–1.30; *p* 0.102) vs. NR (95% CI NR–NR) for HvPD (HR 0.23; 95% CI 0.06–0.88; *p* 0.032). There was no difference between LvPD and DC (HR 1.21; 95% CI 0.20–7.28; *p* 0.838). In patients with metastatic RCC, the second or later line of nivolumab treatment may lead to a deceleration in TGR resulting in an improved survival outcome similar to that observed in patients experiencing tumor regression. In this subgroup, especially in the presence of a clinical benefit, continuing the treatment beyond progression can be recommended.

## 1. Introduction

Renal cell carcinoma (RCC) accounts for about 3% of all malignant tumors worldwide, with an increasing incidence on average of 0.6% each year and about 73,000 new estimated cases in the USA in 2020 [1]. In addition, 25–30% of patients present distant metastases at diagnosis and about 20–40% of patients treated with radical surgery will relapse over time [2] with a 5-year survival rate of approximately 11% for advanced disease [3]. Histologically, RCC can be divided into clear cell RCC (ccRCC), that represents the vast majority accounting for about 75–85% of RCC, and non-clear cell RCC (nccRCC) [4,5]. Of particular importance are prognostic factors able to stratify patients in the metastatic setting into subgroups with a different survival range. The International Metastatic RCC Database Consortium (IMDC) Risk Score elaborated by Heng et al. is the most used prognostic tool [6,7].

The treatment scenario of metastatic RCC has drastically changed in recent years, with the advent of immunotherapy. Over the last decades, the RCC treatment strategy has seen the evolution into three different eras: Firstly the “cytokine era”, then “the targeted therapy era”, and now it is living its third revolution leading to the “golden era” for the management of metastatic RCC with the development of immunotherapy [8].

Since 2015, with the publication of the CheckMate−025 trial, immune-checkpoint inhibitors (ICIs), either alone or in combination with other compounds, are constantly enriching the treatment scenario, with a drastic change of patients’ outcomes. ICIs are able to restore the immune response against tumor cells through the inhibition of specific immune-checkpoint receptors or ligands and consist of programmed death receptor 1/programmed death receptor ligand 1 (PD−1/PD-L1) inhibitors and cytotoxic T-lymphocyte-associated protein 4 (CTLA−4) inhibitors. The CheckMate−025 phase III trial randomized 821 patients, previously treated with at least one antiangiogenic treatment, to receive either nivolumab, a monoclonal antibody IgG4 anti-PD−1 (3 mg per kilogram of body weight intravenously every 2 weeks) or everolimus (10 mg orally once daily) [9,10]. Nivolumab outperformed everolimus in terms of OS (primary endpoint), PFS, and ORR.

ICIs have also changed the first line treatment of metastatic RCC. In recent years, several immune-combination approaches (nivolumab plus ipilimumab [11,12], pembrolizumab plus axitinib [13], avelumab plus axitinib [14], and nivolumab plus cabozantinib [15]) have demonstrated to improve patients’ outcomes compared with sunitinib alone.

The benefit from immunotherapy is difficult to capture with the currently available assessment criteria, considering that the mechanism of action of ICIs is based on a stimulation of the immune system that could result in unusual radiological patterns of response. The current standard response evaluation in clinical trial is response evaluation criteria in solid tumors (RECIST), that has been validated for classic anticancer treatments [16]. With the advent of immunotherapy, that is characterized by a different pattern of response from classic chemotherapy or other anticancer compounds, novel radiological criteria for response have been developed (immune-RECSIT [iRECIST]) [17]. RECIST criteria are based on the evaluation of the change in tumor burden in terms of objective tumor response for target lesions (CR; partial responses, PR; stable disease, SD; progressive disease, PD). RECIST criteria take into account the minimum size of measurable lesions, number of lesions to follow (up to 10; a maximum of five per organ site), sums of target lesions diameters, and appearance of new lesions [16]. The mechanism of action of ICIs is based on immune and T-cell activation that can lead to unusual patterns of response. The consequent radiologic evaluation can be altered considering that enhanced immune infiltration can lead to more pronounced tumor dimensions. The iRECIST criteria are based on RECIST 1.1. and consist of: Immune complete response (iCR), immune partial response (iPR), unconfirmed progressive disease (iUPD), and confirmed progressive disease (iCPD) [17]. The objective response is mainly assessed as RECIST 1.1, but iRECIST take into account the concept of resetting the bar if RECIST 1.1 progression is followed at the next assessment by tumor shrinkage. In this context, iUPD requires confirmation and if progression is not confirmed, the bar is reset and iUPD needs to occur again and then be confirmed with an evidence of tumor growth at the next assessment. This evaluation is of critical importance considering that ICIs can also lead to atypical patterns of response, such as hyperprogression or pseudoprogression. Pseudoprogression consists of an initial increase in tumor burden followed by a response to therapy [18,19], while hyperprogression is defined as a tumor growth rate (TGR) that was at least 2-fold greater in patients with disease progression during immunotherapy [20]. Pseudoprogression has been described in 7–8% of RCC [18,19], while hyperprogression is an exceedingly rare event in RCC patients [21,22]. TGR is a dynamic estimation that takes into account the kinetics of tumor burden evolution during a treatment, considering the changes of tumor volume between imaging assessments [23,24,25]. Tumor growth kinetics have been explored for treatment response in multiple types of tumor, including non-small cell lung cancer (NSCLC), RCC during TKI treatment, as well as head and neck tumors [20,26,27]. Considering the different patterns of response to immunotherapy, the RECIST evaluation of response might not be enough to entirely catch the effect of immune checkpoint inhibitors. In addition to this radiologic assessment, the kinetics of tumor volume changes could be an interesting tool to evaluate immunotherapy activity.

We performed a retrospective monocentric study to explore the impact of TGR change after nivolumab administration as second or later line of treatment in patients with metastatic RCC. Our hypothesis was that TGR based on CT measurements could provide clinically relevant information in addition to the RECIST response assessment and could be associated with clinical outcomes in patients with metastatic RCC receiving nivolumab.

## 2. Material and Methods

### 2.1. Patient Population

Data were retrospectively collected from all the consecutive eligible patients with metastatic RCC treated with nivolumab in second or later lines of therapy at Sant’Orsola-Malpighi Hospital from 1 January 2015 to 30 September 2020.

To be eligible, patients had to present the following criteria of inclusion:

- Eighteen years or older;
- Histological confirmation of RCC (both clear cell and non-clear cell);
- Stage IV disease;
- Available computer tomography (CT) scans for radiological evaluation (within 30 days from treatment onset, and 2–4 months before and after);
- Patients could have received previous lines of therapy with tyrosine kinase inhibitors, but could not have received a previous immune-checkpoint inhibitor.

Other data of interest were: Histological subtype, gender, presence of liver metastases, presence of bone metastases, number and type of systemic treatments received previous to or following nivolumab, IMDC risk group at initiation of the first line treatment.

Patients without available CT scans at each time point were excluded from the study. 

The end of the follow-up period was 15 September 2020.

The study was approved by the local Institution Review Board (Comitato Etico Indipendente, S.Orsola Malpighi Hospital, Bologna) and was conducted in accordance with the principles of the Declaration of Helsinki (6th revision, 2008).

### 2.2. Tumor Volume and Tumor Growth Rate Calculation

Philips IntelliSpace Portal v. 8.0 (Philips Medical System, Eindhoven, The Netherlands) CT software was used to calculate the tumor volume. The iRECIST criteria were used to identify target lesions. Three assessments of tumor volume for each patient were needed to be included in the final analysis. We identified the CT immediately before the start of nivolumab with TC0, while TC−1 was the CT scan performed 8–12 weeks before. TC + 1 was the first assessment performed 8–12 weeks after the start of nivolumab. Considering the exponential growth pattern that regulates cancer cells’ tumor growth, we calculated the tumor volume before the start of nivolumab at time *t* (expressed in days at tumor assessment) with the formula V(t) = V_0_ e^(TG1t)^. V_0_ is the volume at time T_0_, V(t) the volume at time *t*, TG1 the growth rate before the start of nivolumab. Subsequently, we calculated an approximation of the volume at the exact time that nivolumab was started (V_i_). TGR1 and TGR2 represent respectively the percentage increase in tumor volume before and during immunotherapy and were calculated with the following formulas: TGR1 = 100 (e^TG1 – 1^), TGR2 = 100(e^TG2 – 1^). Then, TGR1 and TGR2 were compared for each patient and the resulting data allowed dividing the patients into three groups:

- Disease control (DC): Patients that did not present PD;
- Lower velocity PD (LvPD): Progressive disease but TGR2 was lower than TGR1;
- Higher velocity PD (HvPD): Progressive disease but TGR2 was higher than TGR1.

### 2.3. Statistical Analysis

We used summary statistics to categorize clinical and pathological information. The Fisher exact test and ANOVA were used to analyse associations between DC, LvPD or HvPD and categorical or continuous variables, respectively. The median follow-up was calculated with the reverse Kaplan-Meier method and OS was estimated using the Kaplan-Meier method. The Univariate Cox proportional hazards regression model was used to estimate the HR. All the p-values were two-sided. To reach a statistical significance, the p-value needed to be less than 0.05.

A statistical analysis was performed using the Statistical Package for the Social Sciences (SPSS) program version 25.0 (IBM, Armonk, NY, USA).

## 3. Results

Twenty-seven patients were retrospectively enrolled. The patients’ baseline characteristics are summarized in Table 1.

Of the patients included, 10 were categorized as HvPD, eight as LvPD, and nine as DC. The baseline characteristics were well balanced among the categories, except for the number of prior therapies since the HvPD group presented a higher percentage of patients pre-treated with three lines of therapy, while in LvPD and DC groups patients were treated with a maximum of two previous lines of treatment and the majority received only one line (*p* = 0.05). Moreover, patients in the LvPD tended to be older as compared to patients in other groups (*p* 0.063). Differently, patients in the DC group appear to be younger than the other groups: This could influence the ECOG PS status considering that younger people may be more fit.

The median follow-up was 34.9 months (95% CI 12.6–57.2). In the overall population, the median OS was 23.9 months (95% CI 14.5–33.2). In the overall population, the median age was 56 years, 66% were male. The majority (81%) had clear cell RCC. With regards to IMDC risk groups, 41% were in the favorable, 48% in the intermediate, and 11% in the poor risk group. All the patients previously received antiangiogenic therapies with sunitinb, pazopanib, cabozantinib or tivozanib. The previous number of therapies are reported in Table 1. Overall, 12 patients received subsequent lines of therapy after progressing to nivolumab, nine did not receive any other treatment, and six were still receiving nivolumab at the time of last data collection. Of these, only one patient continued nivolumab beyond PD (LvPD group). The distribution of subsequent therapies among the three groups of interest was as follows: In the HvPD group, zero patients were continuing nivolumab, four received other therapies, and six did not. In the LvPD group, one patient was still receiving nivolumab, four received further lines of treatment, and three definitively interrupted the treatment. In the DC group, five patients were still receiving nivolumab, four underwent other lines of therapy, and zero did not receive any other treatment.

**Figure 1:**
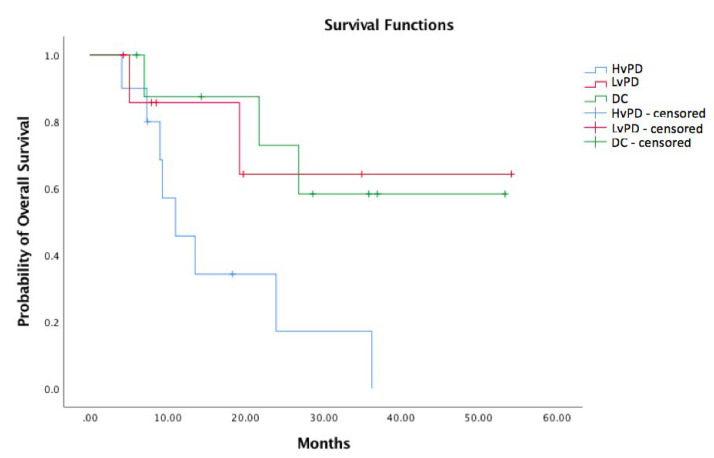
Overall survival according to the tumor growth rate. HvPD: Higher velocity PD (blue); LvPD: Lower velocity PD (red); DC: Disease control (green).

The median OS for the DC group was NR (not reached) (95% CI NR–NR) (reference) vs. NR (95% CI NR-NR) for LvPD (HR 0.27; 95% CI 0.06–1.30; *p* 0.102) vs. 11.0 (95% CI 5.0–17.0) for HvPD (HR 0.23; 95% CI 0.06–0.88; *p* 0.032) (Figure 1). There was no difference between LvPD and DC (HR 1.21; 95% CI 0.20–7.28; *p* 0.838, log-rank).

**Figure 2:**
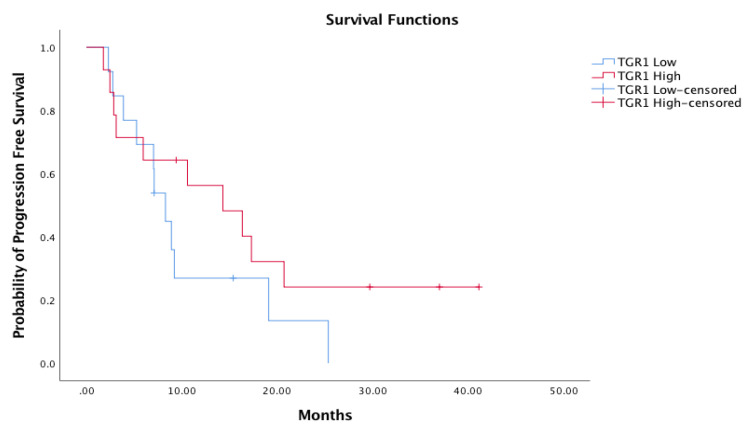
Correlation between TGR1 and PFS. TGR1 low: TGR1 lower than the median. TGR1 high: TGR1 higher than the median.

We also evaluated if TGR1 could be a predictive factor of response to nivolumab. Our hypothesis was that patients rapidly progressing on an antiangiogenic treatment could have better outcomes with the different mechanism of action of immunotherapy. The correlation of TGR1 with PFS was analyzed by dichotomizing using the TGR1 median value (0.00704) as a cut-off. Patients with TGR1 lower than the median (TGR1 low) had a median PFS of 8.2 months (5.2-NA) vs. 14.3 months of those with TGR1 greater than the median (TGR1 high) (5.9-NA) (HR 0.59, CI 95% 0.24–1.4, *p* 0.229), thus TGR1 did not result in statistically influencing PFS on the nivolumab treatment (Figure 2).

## 4. Discussion

Our study aimed at measuring TGR variations in addition to the RECIST 1.1 assessment to evaluate a possible correlation with clinical outcome. The TGR change during the nivolumab treatment was correlated with survival and allowed us to identify two different patterns of progression according to the slope of TGR curve at the first CT scan assessment: One group presented disease progression with no deceleration of tumor growth, and one with progressive disease but presented deceleration of TGR. The third group (DC) was composed of patients responding or having stable disease to nivolumab. There were no statistically significant differences in median OS between the LvPD and DC group (HR 1.21; 95% CI 0.20–7.28; *p* 0.838). Thus, in our analysis, patients that present a decreased TGR (LvPD) seem to have similar outcomes of those responding to nivolumab (DC). This finding seems to suggest that the nivolumab treatment may benefit RCC patients, besides yielding tumor regression or stabilization, also, in some cases, by slowing down the tumor growth rate. This observation has important consequences, since patients with progressive disease are usually considered as treatment failures and are generally not allowed to continue on immunotherapy treatment while, based on our results, those with LvPD, similarly to those having DC, are indeed benefiting from the treatment which should not therefore be stopped. 

We enrolled 27 patients with well-balanced baseline characteristics in terms of histology, sarcomatoid component, Eastern Cooperative Oncology Group Performance Status (ECOG PS), IMDC risk group, presence of liver, and bone metastases.

The standard method for response evaluation in clinical trial is based on the RECIST criteria, that estimate tumor burden based on the sum of the diameters of target lesions and their variation during a treatment. Nonetheless, RECIST present several limitations, mostly related to the unidimensional evaluation of lesions and the lack of dynamic information on tumor kinetics. Immunotherapy is often correlated to scant results in terms of objective response (CR, PR) despite great benefits in terms of OS. Thus, a dynamic evaluation of tumor burden could be helpful to assess the real value of immunotherapy.

In assessing treatment response, RECIST might not be enough to catch the real effect of ICI. In fact, immunotherapy compounds could require a wider time window to show their benefit in terms of objective response, as seen in many trials investigating ICI in RCC. Achieving an objective response, in terms of CR or PR, is not the only factor to consider when choosing a treatment strategy, looking at the benefit in terms of OS demonstrated with immunotherapy treatments. The evaluation of real progressive disease and the choice to change the line of therapy is extremely difficult in advanced lines of treatment that leave our patients with limited active therapies at disposal. In some cases, continuing a treatment beyond a RECIST defined radiologic progression could be an option in the presence of clinical benefit. Distinct patterns of radiologic response to ICIs have been reported in RCC patients [28]. Tumor flare or pseudoprogression could result in early discontinuation of a treatment before it shows its true activity, even more if it is associated with a clinical benefit and it is well tolerated. Continuing the same treatment beyond radiological progression is a therapeutic strategy used in many types of tumors when the patient is experiencing clinical benefit, maintains a good performance status even in the presence of PD, and does not present symptomatic or critical progression in delicate sites of metastasis [29,30].

The subgroup analysis of the CheckMate−025 and a phase II study of patients with RCC undergoing the nivolumab treatment showed that continuing the treatment beyond progression could lead to tumor reduction or lesions stabilization [31,32]. In the subgroup analysis of the CheckMate−025, 153 (48%) patients of the 316 who progressed by the RECIST criteria were treated with nivolumab beyond the first progression [31]. Of these, 13% had a subsequent 30% tumor burden reduction. Of note, clinical characteristics were guided to the decision to continue the treatment beyond progression: Better Karnofsky performance status, less deterioration in Karnofsky performance status, shorter time to response, lower incidence of new bone lesions, and improved quality of life.

TGR has also been evaluated during the target therapy treatment in metastatic RCC. Targeted therapies are often correlated to a stable disease as best overall response rather than a tumor shrinkage. Accordingly, pseudoprogressions during targeted agents’ therapy are possible [33,34,35]. Consequently, TGR could be useful in addition to the RECIST criteria in the response assessment to targeted agents [36]. An integrated analysis of the TARGET and RECORD phase 3 trial data showed that TGR is independently associated with PFS and OS in patients with metastatic RCC treated with everolimus or sorafenib presenting RECIST progression [26]. Moreover, the TGR evaluation allowed identifying a persistence of antitumor activity of sorafenib in patients presenting with RECIST progression. 

Some evidences on TGR as a tool for response evaluation for the ICI treatment have been published, but only one focused on metastatic RCC patients. Mazza et al. reported the results of a central radiological review of 45 patients treated with nivolumab at Institut Gustave Roussy within the GETUG-AFU 26 NIVOREN (EudraCT 2015–004117) study [37]. Fifty-nine percent of patients presented a decrease in TGR at first assessment compared with the TGR pre-treatment. Seventeen percent of patients had an initial increase in TGR at first assessment with a subsequent decrease in TGR at second assessment. With regards to NSCLC, Berge et al. evaluated differences in TGR before and after the start of the anti-PD−1 treatment in 58 patients [38]. Thirty-seven patients presented a deceleration of TGR after initiation of therapy with a significant difference in median OS (18.0 vs. 6.0 months, HR 0.35, 95% CI 0.18–0.71) between these patients and those showing an acceleration of TGR. Another retrospective study on 73 advanced NSCLC patients receiving anti-PD−1/PD-L1 monotherapy confirmed these results: Higher TGR at first evaluation was correlated with inferior outcomes (higher TGR vs. lower HR 2.74, 95% CI 1.34–5.61, *p* = 0.006).

In our study, a decreased TGR during the treatment with nivolumab resulted in being associated with improved survival. Thus, patients on RECIST defined that progression to nivolumab could be divided into two categories with different outcomes: The ones with progressive disease presenting an acceleration in tumor growth and the ones in which, despite progression of disease, tumor growth velocity is decreased. In this latter subgroup, continuing nivolumab beyond radiological progression could be an option in the case of clinical benefit, given the option of pseudoprogression with the hope of a response in the following assessment or given the survival benefit associated with a decelerated tumor growth. TGR could be a helpful factor to take into account during the ICIs treatment in addition to the RECIST criteria since it allows a dynamic assessment of the tumor behavior and its kinetics. It has to be kept in mind that the total number of patients included in the study, especially considering that they are divided into three groups, make the statistical analysis of low strength. In addition, the study should be expanded to have more meaningful data.

The strengths of our work are that a dedicated software and a specific formula have been used to estimate TGR and to approximate the tumor growth in the interval between the TC0 and the actual start of nivolumb treatment. By this, a more accurate measurement of TGR on nivolumab was performed, increasing the reliability of our data. Furthermore, as TGR is a relatively novel approach to assess tumor kinetics on treatment [30], there is scant preliminary data about this approach in patients with RCC on immunotherapy. In this scenario, the results of our work appear as a novel approach that could be helpful in everyday clinical practice.

Several limitations could affect the results of our study. First of all, our analysis is a single-center retrospective study of a cohort of patients with metastatic RCC. Secondly, the limited number of patients is a relevant factor that could impair the strength of our results. Widening our casistic is one the main perspectives of our future line of work on this project. Moreover, a control group not receiving immunotherapy could consolidate our results.

## 5. Conclusions

The RECIST criteria present some concerns in evaluating the real benefit from an immunotherapy approach in RCC. The tumor growth rate evaluation could be helpful in distinguishing, among patients with PD on immune-checkpoint inhibitors, those that may still benefit from continuing the treatment beyond RECIST progression. A deceleration in TGR, despite RECIST PD, is associated with a better survival outcome in patients receiving nivolumab as an advanced line of therapy for metastatic RCC. In this subgroup, if a clinical benefit is still experienced by the patient, continuing immunotherapy beyond PD can be recommended. Further prospective studies are warranted to validate these retrospective findings.

## Figures and Tables

**Table 1:** The patients’ baseline characteristics and *p*-value for associations between DC, LvPD or HvPD and categorical or continuous variables. HvPD: Higher velocity PD; LvPD: Lower velocity PD; DC: Disease control; IMDC: International Metastatic RCC Database Consortium; ECOG PS: Eastern Cooperative Oncology Group Performance Status; NA: Not available.

		HvPD (*n* = 10)	LvPD (*n* = 8)	DC (*n* = 9)	All Patients (*n* = 27)	*p*-Value
Age (mean)		57.5	68	44	56	0.063
Sex *n* (%)	Male	7 (70%)	5 (62.5%)	6 (66.5%)	18 (66%)	0.945
	Female	3 (30%)	3 (37.5%)	3 (33.5%)	9 (33%)	
Histology *n* (%)	Clear cell	8 (80%)	7 (87.5%)	7 (78%)	22 (81%)	0.866
	Non-clear cell	2 (20%)	1 (12.5%)	2 (22%)	5 (19%)	
Sarcomatoid component *n* (%)	Yes	2 (20%)	3 (37.5%)	4 (44.4%)	9 (33%)	0.959
	No	2 (20%)	3 (37.5%)	3 (33.3%)	8 (30%)	
	NA	6 (60%)	2 (25%)	2 (22.2%)	10 (37%)	
IMDC risk groups *n* (%)	Good	6 (60%)	3 (37.5%)	2 (22.5%)	11 (41%)	0.398
	Intermediate	4 (40%)	4 (50%)	5 (55%)	13 (48%)	
	Poor	0 (0%)	1 (12.5)	2 (22.5%)	3 (11%)	
ECOG PS *n* (%)	0	6 (60%)	4 (50%)	6 (66.5%)	16 (59%)	0.782
	1	4 (40%)	4 (50%)	3 (33.5%)	11 (41%)	
Number of prior therapies *n* (%)	1	2 (20%)	5 (62.5%)	6 (66.5%)		0.05
	2	4 (40%)	3 (37.5%)	3 (33.5%)		
	3	4 (40%)	0 (0%)	0 (0%)		
Number of subsequent lines *n* (%)	0	6 (60%)	4 (50%)	5 (56%)		0.380
	1	3 (30%)	3 (37.5%)	3 (33%)		
	2	1 (10%)	1 (12.5%)	1 (11%)		
Bone metastases *n* (%)	Yes	4 (40%)	3 (37.5%)	3 (33.5%)	10 (37%)	0.955
	No	6 (60%)	5 (62.5%)	6 (66.5%)	17 (63%)	
Liver metastases *n* (%)	Yes	2 (20%)	3 (37.5%)	3 (33.5%)	8 (29.6%)	0.690
	No	8 (80%)	5 (62.5%)	6 (66.5%)	19 (70.4%)	

## Data Availability

The data presented in this study are available in database at Medical Oncology, IRCCS Azienda Ospedaliero Universitaria—Bologna.

## References

[1] Siegel R.L., Miller K.D., Jemal A. (2020). Cancer statistics, 2020. CA Cancer J. Clin..

[2] Janzen N.K., Kim H.L., Figlin R.A., Belldegrun A.S. (2003). Surveillance after radical or partial nephrectomy for localized renal cell carcinoma and management of recurrent disease. Urol. Clin. N. Am..

[3] NCCN Clinical Practice Guidelines in Oncology (NCCN Guidelines^®^). Kidney Cancer.

[4] Moch H., Cubilla A.L., Humphrey P.A., Reuter V.E., Ulbright T.M. (2016). The 2016 WHO classification of tumours of the urinary system and male genital organs—part A: Renal, penile, and testicular tumours. Eur. Urol..

[5] Massari F., Di Nunno V., Santoni M., Gatto L., Caserta C., Morelli F., Zafarana E., Carrozza F., Mosca A., Mollica V. (2019). Toward a genome-based treatment landscape for renal cell carcinoma. Crit. Rev. Oncol. Hematol..

[6] Heng D.Y., Xie W., Regan M.M., Warren M.A., Golshayan A.R., Sahi C., Eigl B.J., Ruether J.D., Cheng T., North S. (2009). Prognostic factors for overall survival in patients with metastatic renal cell carcinoma treated with vascular endothelial growth factor-targeted agents: Results from a large, multicenter study. J. Clin. Oncol..

[7] Heng D.Y., Xie W., Regan M.M., Harshman L.C., Bjarnason G.A., Vaishampayan U.N., Mackenzie M., Wood L., Donskov F., Tan M.H. (2013). External validation and comparison with other models of the International Metastatic Renal-Cell Carcinoma Database Consortium prognostic model: A population-based study. Lancet Oncol..

[8] Choueiri T.K., Motzer R.J. (2017). Systemic Therapy for Metastatic Renal-Cell Carcinoma. N. Engl. J. Med..

[9] Motzer R.J., Escudier B., McDermott D.F., George S., Hammers H.J., Srinivas S., Tykodi S.S., Sosman J.A., Procopio G., Plimack E.R. (2015). CheckMate 025 Investigators. Nivolumab versus Everolimus in Advanced Renal-Cell Carcinoma. N. Engl. J. Med..

[10] Motzer R.J., Escudier B., George S., Hammers H.J., Srinivas S., Tykodi S.S., Sosman J.A., Plimack E.R., Procopio G., McDermott D.F. (2020). Nivolumab versus everolimus in patients with advanced renal cell carcinoma: Updated results with long-term follow-up of the randomized, open-label, phase 3 CheckMate 025 trial. Cancer.

[11] Motzer R.J., Tannir N.M., McDermott D.F., Arén Frontera O., Melichar B., Choueiri T.K., Plimack E.R., Barthélémy P., Porta C., George S. (2018). CheckMate 214 Investigators. Nivolumab plus Ipilimumab versus Sunitinib in Advanced Renal-Cell Carcinoma. N. Engl. J. Med..

[12] Motzer R.J., Rini B.I., McDermott D.F., Arén Frontera O., Hammers H.J., Carducci M.A., Salman P., Escudier B., Beuselinck B., Amin A. (2019). CheckMate 214 investigators. Nivolumab plus ipilimumab versus sunitinib in first-line treatment for advanced renal cell carcinoma: Extended follow-up of efficacy and safety results from a randomised, controlled, phase 3 trial. Lancet Oncol..

[13] Rini B.I., Plimack E.R., Stus V., Gafanov R., Hawkins R., Nosov D., Pouliot F., Alekseev B., Soulières D., Melichar B. (2019). KEYNOTE-426 Investigators. Pembrolizumab plus Axitinib versus Sunitinib for Advanced Renal-Cell Carcinoma. N. Engl. J. Med..

[14] Motzer R.J., Penkov K., Haanen J., Rini B., Albiges L., Campbell M.T., Venugopal B., Kollmannsberger C., Negrier S., Uemura M. (2019). Avelumab plus Axitinib versus Sunitinib for Advanced Renal-Cell Carcinoma. N. Engl. J. Med..

[15] Choueiri T.K., Powles T., Burotto M., Escudier B., Bourlon M.T., Zurawski B., Oyervides Juárez V.M., Hsieh J.J., Basso U., Shah A.Y. (2021). CheckMate 9ER Investigators. Nivolumab plus Cabozantinib versus Sunitinib for Advanced Renal-Cell Carcinoma. N. Engl. J. Med..

[16] Eisenhauer E.A., Therasse P., Bogaerts J., Schwartz L.H., Sargent D., Ford R., Dancey J., Arbuck S., Gwyther S., Mooney M. (2009). New response evaluation criteria in solid tumours: Revised RECIST guideline (version 1.1). Eur. J. Cancer.

[17] Seymour L., Bogaerts J., Perrone A., Ford R., Schwartz L.H., Mandrekar S., Lin N.U., Litière S., Dancey J., Chen A. (2017). iRECIST: Guidelines for response criteria for use in trials testing immunotherapeutics. Lancet Oncol..

[18] Queirolo P., Spagnolo F. (2017). Atypical responses in patients with advanced melanoma, lung cancer, renal-cell carcinoma and other solid tumors treated with anti-PD-1 drugs: A systematic review. Cancer Treat. Rev..

[19] Chiou V.L., Burotto M. (2015). Pseudoprogression and Immune-Related Response in Solid Tumors. J. Clin. Oncol..

[20] Champiat S., Dercle L., Ammari S., Massard C., Hollebecque A., Postel-Vinay S., Chaput N., Eggermont A., Marabelle A., Soria J.C. (2017). Hyperprogressive Disease Is a New Pattern of Progression in Cancer Patients Treated by Anti-PD-1/PD-L1. Clin. Cancer Res..

[21] Hwang I., Park I., Yoon S.K., Lee J.L. (2020). Hyperprogressive Disease in Patients With Urothelial Carcinoma or Renal Cell Carcinoma Treated With PD-1/PD-L1 Inhibitors. Clin. Genitourin. Cancer.

[22] Soria F., Beleni A.I., D’Andrea D., Resch I., Gust K.M., Gontero P., Shariat S.F. (2018). Pseudoprogression and hyperprogression during immune checkpoint inhibitor therapy for urothelial and kidney cancer. World J. Urol..

[23] Gomez-Roca C., Koscielny S., Ribrag V., Dromain C., Marzouk I., Bidault F., Bahleda R., Ferté C., Massard C., Soria J.C. (2011). Tumour growth rates and RECIST criteria in early drug development. Eur. J. Cancer.

[24] Ferté C., Fernandez M., Hollebecque A., Koscielny S., Levy A., Massard C., Balheda R., Bot B., Gomez-Roca C., Dromain C. (2014). Tumor growth rate is an early indicator of antitumor drug activity in phase I clinical trials. Clin. Cancer Res..

[25] Le Tourneau C., Servois V., Diéras V., Ollivier L., Tresca P., Paoletti X. (2012). Tumour growth kinetics assessment: Added value to RECIST in cancer patients treated with molecularly targeted agents. Br. J. Cancer.

[26] Ferté C., Koscielny S., Albiges L., Rocher L., Soria J.C., Iacovelli R., Loriot Y., Fizazi K., Escudier B. (2014). Tumor growth rate provides useful information to evaluate sorafenib and everolimus treatment in metastatic renal cell carcinoma patients: An integrated analysis of the TARGET and RECORD phase 3 trial data. Eur. Urol..

[27] Saâda-Bouzid E., Defaucheux C., Karabajakian A., Coloma V.P., Servois V., Paoletti X., Even C., Fayette J., Guigay J., Loirat D. (2017). Hyperprogression during anti-PD-1/PD-L1 therapy in patients with recurrent and/or metastatic head and neck squamous cell carcinoma. Ann. Oncol..

[28] de Velasco G., Krajewski K.M., Albiges L., Awad M.M., Bellmunt J., Hodi F.S., Choueiri T.K. (2016). Radiologic Heterogeneity in Responses to Anti-PD-1/PD-L1 Therapy in Metastatic Renal Cell Carcinoma. Cancer Immunol. Res..

[29] Reinhorn D., Jacobi O., Icht O., Dudnik E., Rotem O., Zer A., Goldstein D.A. (2020). Treatment beyond progression with immune checkpoint inhibitors in non-small-cell lung cancer. Immunotherapy.

[30] Masi G., Salvatore L., Boni L., Loupakis F., Cremolini C., Fornaro L., Schirripa M., Cupini S., Barbara C., Safina V. (2015). Continuation or reintroduction of bevacizumab beyond progression to first-line therapy in metastatic colorectal cancer: Final results of the randomized BEBYP trial. Ann. Oncol..

[31] Escudier B., Motzer R.J., Sharma P., Wagstaff J., Plimack E.R., Hammers H.J., Donskov F., Gurney H., Sosman J.A., Zalewski P.G. (2017). Treatment Beyond Progression in Patients with Advanced Renal Cell Carcinoma Treated with Nivolumab in CheckMate 025. Eur. Urol..

[32] George S., Motzer R.J., Hammers H.J., Redman B.G., Kuzel T.M., Tykodi S.S., Plimack E.R., Jiang J., Waxman I.M., Rini B.I. (2016). Safety and Efficacy of Nivolumab in Patients with Metastatic Renal Cell Carcinoma Treated Beyond Progression: A Subgroup Analysis of a Randomized Clinical Trial. JAMA Oncol..

[33] Rosen M.A. (2010). Use of modified RECIST criteria to improve response assessment in targeted therapies: Challenges and opportunities. Cancer Biol. Ther..

[34] Inman B.A., George D.J. (2011). Is tumor response important for renal carcinoma?. Eur. Urol..

[35] Santoni M., Buti S., Conti A., Porta C., Procopio G., Sternberg C.N., Bracarda S., Basso U., De Giorgi U., Rizzo M. (2015). Prognostic significance of host immune status in patients with late relapsing renal cell carcinoma treated with targeted therapy. Target Oncol..

[36] Stein W.D., Wilkerson J., Kim S.T., Huang X., Motzer R.J., Fojo A.T., Bates S.E. (2012). Analyzing the pivotal trial that compared sunitinib and IFN-α in renal cell carcinoma, using a method that assesses tumor regression and growth. Clin. Cancer Res..

[37] Mazza C., Arfi-Rouche J., Koscielny S., Caramella C., Lahmar J., Escudier B.J., Albiges L. (2017). Effect of nivolumab on tumor growth rate (TGR) in metastatic renal cell carcinoma (mRCC). J. Clin. Oncol..

[38] Berge D.T., Hurkmans D.P., den Besten I., Kloover J.S., Mathijssen R., Debets R., Smit E.F., Aerts J. (2019). Tumour growth rate as a tool for response evaluation during PD-1 treatment for non-small cell lung cancer: A retrospective analysis. ERJ Open Res..

